# Identifying the Need for Prehabilitation in Cancer Patients Undergoing Nephrectomy or Nephroureterectomy

**DOI:** 10.3390/cancers17172939

**Published:** 2025-09-08

**Authors:** Bente Thoft Jensen, Peter Blak Hjort, Kathrine Melchiorsen, Henriette Vind Thaysen, Ida Larsen, Mai Lorenzen, Rikke Knudsen, Anna K. Keller

**Affiliations:** 1 Department of Urology, Aarhus University Hospital, 8200 Aarhus, Denmarkidasoendergaard@hotmail.com (I.L.); mai.lor@rm.dk (M.L.); rikkknud@rm.dk (R.K.); anna.keller@clin.au.dk (A.K.K.); 2 National Center for Research in Cancer Surgery (ACROBATIC), Aarhus University, 8200 Aarhus, Denmark; henrthay@rm.dk; 3 Department of Clinical Medicine, Aarhus University, 8200 Aarhus, Denmark; 4 Department of Colorectal Surgery, Aarhus University Hospital, 8200 Aarhus, Denmark

**Keywords:** prehabilitation, renal cancer, upper urinary tract urothelial cancer, nephrectomy, nephroureterectomy, physical function, recovery

## Abstract

Background: The potential benefits of prehabilitation in patients undergoing surgery for renal cell carcinoma (RCC) or upper-tract urothelial carcinoma (UTUC) remain unknown. The aim was to evaluate physical function and baseline characteristics over the course of treatment to identify a potential need for prehabilitation. Methods: In this prospective observational study, 62 patients were enrolled—31 undergoing nephrectomies for RCC and 31 undergoing nephroureterectomy for UTUC. Baseline assessments included nutritional screening (NRS 2002), frailty (Clinical Frailty Scale), hemoglobin and iron levels, smoking status, and physical function using the Six-Minute Walk Test (6MWT) and the 30-Second Sit-to-Stand Test (30STS). Functional tests were repeated at hospital discharge and at two-week postoperative follow-up visit. Results: At baseline, 45% of RCC and 68% of UTUC patients were at nutritional risk. Preoperative frailty was present in 20% of the cohort, and 53% had anemia. Functional impairment below the lower limit values (LLVs) was observed in 16% for the RCC and 36% of the UTUC, assessed by 6MWT. The 30 STS revealed that 58% of RCC and 42% of UTUC were below LLV. At discharge, impairment peaked, with 59% and 82% of patients being below the LLVs, respectively. Functional performance partially recovered at follow-up but did not return to baseline levels. Conclusions: Preoperative nutritional deficits, anemia, and functional impairment are prevalent in patients undergoing nephrectomy or nephroureterectomy. A marked postoperative functional decline was identified postoperatively supporting a potential need for early individualized prehabilitation strategies to improve recovery in patients undergoing kidney cancer surgery.

## Simple Summary

This study investigated the potential need for prehabilitation, describing baseline characteristics and functional outcomes in patients undergoing surgery for renal cell carcinoma (RCC) or upper-tract urothelial carcinoma (UTUC). A total of 62 patients were prospectively enrolled and assessed for modifiable preoperative risk factors and physical performance using the Six-Minute Walk Test and the 30-Second Sit-to-Stand Test. Approximately 20% of patients were identified as frail preoperatively. A decline in physical function at discharge was seen in both groups, with partial recovery observed at two-week follow-up. Nutritional risk and smoking were more prevalent in the UTUC group. These findings highlight the importance of early identification of prehabilitation targets to optimize perioperative care in kidney cancer surgery.

## 1. Introduction

Prehabilitation can be defined as any preoperative intervention aiming to improve a patient’s functional status in efforts to minimize postoperative impairments, surgical complications and maximize outcomes [1]. Any preoperative condition that prevents a patient from tolerating the upcoming physiological stress of surgery augments the catabolic response to stress and increases the risk for poor surgical outcomes and prolonged recovery [2,3]. Prehabilitation interventions that include, e.g., replacement interventions for tobacco, physical exercise, and nutrition components can be implemented before surgery to strengthen physiological reserve and enhance physical function, which, in turn, supports recovery through attaining surgical resilience [4,5,6]. Prehabilitation complements enhanced recovery after surgery (ERAS) care to achieve optimal patient outcomes, as recovery is not a passive process; rather, it begins preoperatively [7]. Thus, prehabilitation is considered to have both a prevention and a health promotion aspect, with the goal of enhancing individual functional status to support the patient to better withstand the upcoming surgical stress response, optimize physical capacity, and reduce modifiable risk factors prior to cancer surgery [1,8].

Renal cell cancer (RCC) is one of the most prevalent urological malignancies, with its incidence nearly doubling since the 1970s [9]. RCC is most often discovered accidentally before the patient has symptoms, whereas upper urinary tract urothelial cell carcinoma (UTUC) is less prevalent and most often found in older and symptomatic patients [10]. Surgery remains the curative treatment through radical nephrectomy and nephroureterectomy, respectively.

Surgical cancer patients often present with frailty; decreased homeostatic reserve; diminished physical and psychological strength; reduced weight; and sarcopenia, all of which portend to an increased risk for postoperative morbidity and prolonged hospitalizations [11]. These deficits are repeatedly observed in patients with urologic malignancies, therefore promoting investigating the clinical benefits of surgical prehabilitation beyond advanced bladder cancer pathways [8,12]. While recent studies suggest that prehabilitation may improve outcomes in patients undergoing kidney transplantation [13,14], the role of prehabilitation and its efficacy in individuals undergoing nephrectomy or nephroureterectomy for malignancy remains uncertain. Preoperative functional status and nutritional deficits are prevalent in patients undergoing cancer surgery, and these deficits are associated with a transient postoperative functional decline, indicating potential benefit from structured prehabilitation. So far, this association has not been reported in kidney cancer surgery.

This observational cohort study aims to evaluate the role and potential benefits of prehabilitation in patients scheduled for nephrectomy or nephroureterectomy. Specifically, this study seeks to assess changes in functional status from admission to follow-up post-surgery to identify modifiable preoperative risk factors.

## 2. Materials and Methods 

Patients referred to the Department of Urology at Aarhus University Hospital for either nephrectomy due to RCC or nephroureterectomy due to UTUC between 1 February 2023 and 1 February 2024 were included in the study. All patients received verbal and written information regarding the purpose of the study upon consenting to surgery as their treatment modality. Patients not able to perform the functional tests were excluded. Informed consent was obtained from all participants. To identify the potential need for prehabilitation, we employed a composite assessment tool comprising evidence-based prehabilitation parameters developed by the Prehabilitation Section of the Danish National Center for Research in Cancer Surgery (ACROBATIC) [15] reflecting the components in the short geriatric eight question screening tool (G8) developed for elderly patients undergoing cancer surgery including the domains body mass index (BMI), nutrition, cognition, functional level, polypharmacy, age, and self-assed health status [16,17]. Frailty was measured by the Clinical Frailty Screening tool [18], and polypharmacy was defined as using three or more medications at the same time.

In Denmark, nutritional risk assessment prior to cancer surgery is mandated by national health authorities and is conducted using the validated Nutritional Risk Screening tool (NRS-2002), which is integrated into the electronic medical patient record (EMR) [19]. Additional variables collected from the EMR included serum albumin, hemoglobin levels, and serum iron to establish potential anemia.

The core physical function was assessed using the gold-standard measures: 6-Minute Walking Test (6MWT), reflecting physical endurance [20], and the 30-Second Sit-to-Stand test (30STS), reflecting ability to perform activities of daily living [21]. The potential need for prehabilitation was identified by the proportion of patients below predicted lower limit values (LLVs) for 6MWT and 30STS, respectively, using standardized reference equations [21,22,23]. Both tests were adjusted for gender and age. All patients were assessed preoperatively, at discharge, and at follow-up two weeks after discharge by a team of two specialized rehabilitation physiotherapists or a trained research nurse. A baseline assessment of potential risk factors is presented using descriptive statistics. Categorical data is described using proportions and percent and continuous data, with median (Q1 to Q3) values being provided in cases of non-normal distribution. To test for statistical differences in functional status within and between groups, we performed McNemar’s test and conducted pairwise comparisons for each surgical group at each time point. The Statistical management program “R” was used to test for any significant differences. It’s free and widely used in both research and teaching across many disciplines at Aarhus University (DK). *p*-values < 0.05 were considered statistically significant.

## 3. Results

### 3.1. Baseline Characteristics

A total of 78 patients were invited to participate, of whom 62 were enrolled. Eight patients declined due to lack of interest or non-ability to perform the functional tests; six patients were referred to further cancer evaluation, and two missed clinic appointments. The study population was evenly distributed, with 31 patients allocated to the RCC cohort (n_1_) undergoing nephrectomy and 31 allocated to the UTUC cohort (n_2_) undergoing nephroureterectomy (Table 1). 

A higher proportion of males underwent kidney surgery compared to females, and the majority of operations were laparoscopic. Cases of individuals being at nutritional risk, assessed by the NRS 2002 screening tool, were observed in 45% and in 68% of patients in the RCC cohort and the UTUC cohort, respectively. Overall smoking status revealed that 23% of patients were current smokers; however, the prevalence was higher in the UTUC cohort (30% vs. 14%).

No differences were observed between the groups in terms of cognitive status, polypharmacy, or frailty. Nonetheless, approximately 20% of all patients were classified as frail based on the Clinical Frailty Scale. Furthermore, 53% of the total cohort presented with mild to moderate anemia preoperatively, and 44% exhibited low serum iron levels in their baseline bloodwork (Table 1).

### 3.2. Functional Tests

#### 3.2.1. The 6MWT 

**Figure 1:**
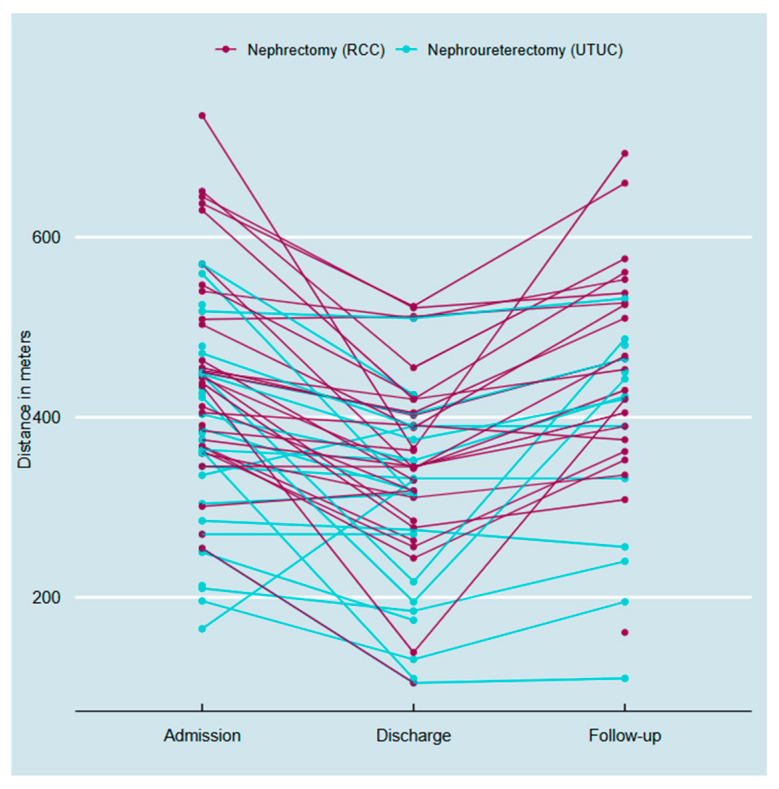
Developments in 6MWT in 31 patients undergoing nephrectomy for RCC and 31 patients undergoing nephroureterectomy at Aarhus University Hospital 2023–2024.

In the RCC cohort (n_1_), 16% of patients exhibited performance below the predicted LLV at baseline. This proportion decreased to 59% at the time of hospital discharge and subsequently increased to 23% at the follow-up assessment. In the UTUC cohort (n_2_), 36% of patients performed below the LLV at baseline. This proportion declined to 50% at discharge and then increased slightly to 43% in the two-week postoperative follow-up (Figure 1). 

Within the nephrectomy group, a significant decline in 6MWT was observed between admission and discharge (*p* < 0.001), along with significant improvement between discharge and follow-up (*p* = 0.031), whereas no significant difference was found between admission and follow-up (*p* = 0.25). In the nephroureterectomy group, no statistically significant differences were observed between admission and discharge (*p* = 0.375), discharge and follow-up (*p* = 0.50), or admission and follow-up (*p* = 1.00).

#### 3.2.2. The 30STS Test

**Figure 2:**
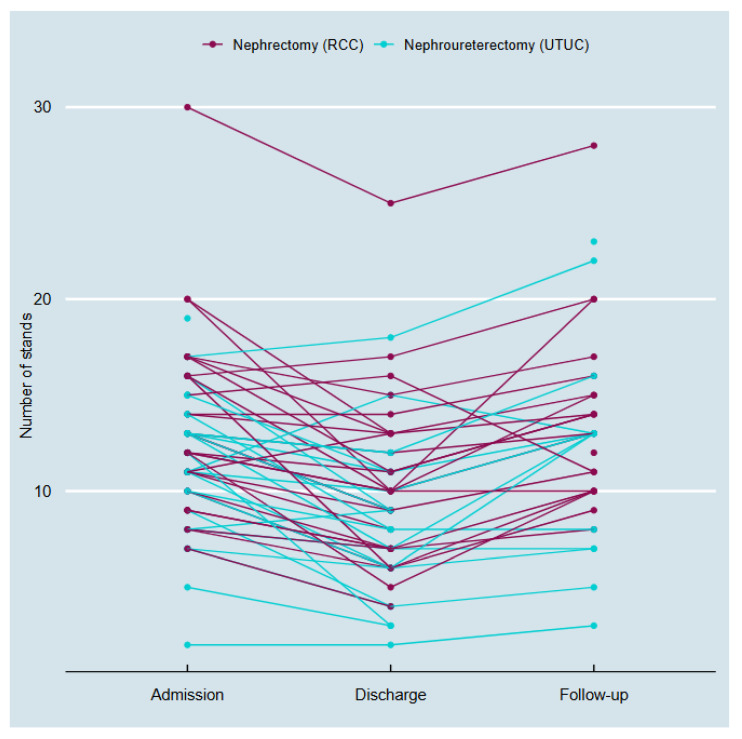
Developments in the 30STS in 31 patients undergoing nephrectomy for RCC and 31 patients undergoing nephroureterectomy for UTUC at Aarhus University Hospital 2023–2024.

A similar trend was observed in the 30STS test. In the RCC cohort (n_1_), 58% of patients performed below the LLV at baseline, decreasing to 76% at discharge and returning to near baseline levels (52%) at follow-up. In the UTUC cohort (n_2_), 42% of patients scored below the LLV at baseline. This decreased to 82% at discharge and subsequently increased to 57% at follow-up (Figure 2). In the nephrectomy group, no statistically significant differences in 30 STS test were observed between admission and discharge (*p* = 0.188), discharge and follow-up (*p* = 0.25), or admission and follow-up (*p* = 1.00). However, in the nephroureterectomy group, a statistically significant difference was observed between admission and discharge (*p* = 0.047), whereas no significant differences were found between discharge and follow-up (*p* = 1.00) or admission and follow-up (*p* = 1.00). 

Between the two surgical groups, no statistically significant differences were observed for any test at any time point.

## 4. Discussion

This is the first study to investigate whether patients undergoing surgery for RCC and UTUC present with modifiable preoperative risk factors at time for surgery and describe changes in physical function over the course of treatment. Nutritional risk was observed in 45% of RCC patients and 68% of UTUC patients (Table 1), and a marked decline in physical performance was documented (Figure 1 and Figure 2). The findings support the expectations of a decline in physical function postoperatively and could indicate a potential need for prehabilitation to counteract this decline [24]. 

Both the 6MWT and 30STS outcomes have shown to be independent predictors of postoperative morbidity and mortality [25,26]. The baseline assessments revealed a high prevalence of functional impairments, particularly in the 30STS-Test, where over half of patients in the RCC group and over 40% in the UTUC group scored below the LLV, which could indicate the potential for prehabilitation interventions to improve functional status before admission to diminish a further decline during admission. However, we found no statistical differences between admission and follow-up within and between the cohorts at any time point.

Regarding the 6MWT a similar trend was observed; 16% and 36% of patients in the RCC and UTUC cohorts, respectively, fell below the LLV for the 6MWT. Within the nephrectomy group, a significant decline was observed between admission and discharge (*p* < 0.001), but the individuals nearly regained baseline functional level at follow-up, although no difference was observed from admission to discharge within or between the cohorts. These data demonstrate that a high proportion of patients present with suboptimal physical function before surgery, which could promote prehabilitation interventions to counteract a further decline and support early recovery. This preoperative vulnerability was further compounded by high rates of patients being at nutritional risk—particularly in the UTUC group (68%)—assessed by the NRS-2002. Although sample size was limited, we tested for differences reaching the LLV value for both the 6MWT and 30STS test for each surgical group, based on nutritional status, but we observed no statistical differences.

These findings align with the limited existing evidence in kidney cancer surgery indicating that RCC and UTUC patients are at heightened nutritional risk and that adjustments may support recovery [24,27,28,29]. Together, impaired physical function and nutritional deficits may represent key targets for potential multimodal prehabilitation interventions, as demonstrated in other cancer types [30,31]. Recently, the importance of physical exercise for restoring physical function after cancer treatment was confirmed in a large randomized controlled trial, which showed a significant improvement in cancer survival [32]. Thus, there is a growing recognition that prehabilitation strategies emphasizing the optimization of functional status prior to surgery may also have a future role in patients undergoing kidney cancer surgery [27,30].

To promote early recovery in kidney cancer surgery, the concept of enhanced recovery after surgery (ERAS) has been implemented over the past two decades, with positive impact on recovery [24,31]. The ERAS concept provides a multimodal, multi-professional approach to control perioperative pathophysiology caused by the surgical stress response, thereby mitigating the risk of organ dysfunction and subsequently enhancing recovery [32]. The onset of this response entails body proteins that can be significantly catabolized and decrease one’s capacity to restore pre-surgery physical function [33]. This was demonstrated in the postoperative assessments, which revealed a significant decline in aerobic capacities measured by the 6MWT and lower limb strength measured by the 30STS test in the nephroureterectomy group (Figure 1 and Figure 2). Functional decline peaked at discharge, where 76–82% of patients fell below LLV on the 30STS, with partial recovery by the two-week follow-up. These data are consistent with prior studies showing early postoperative deconditioning after major uro-oncologic surgery [8,12,34]. Importantly, this transient functional impairment may delay recovery and further support the rationale for early preoperative interventions. Anemia and low iron levels were also frequently observed at baseline, affecting 53% and 44% of patients, respectively (Table 1). Preoperative anemia has been shown to increase surgical risk, lengthen hospital stays, and worsen survival in cancer patients [8]. Further, two studies have shown that preoperative anemia is an independent predictor of disease recurrence and cancer-specific mortality in patients with UTUC undergoing nephroureterectomy [35,36]. Optimizing iron status preoperatively through iron supplementation or erythropoiesis-stimulating agents may be considered as part of a broader prehabilitation strategy.

Frailty was identified in 20% of patients and continues to be a critical factor, given its strong predictive value for postoperative complications—as evidenced across uro-oncology and other surgical specialties [7,37]. A recent review focusing on RCC patients highlights the unmet clinical need for incorporating structured frailty assessments into routine care pathways. In line with our study, a pragmatic strategy involving the Clinical Frailty Scale has been proposed to screen older patients and guide tailored perioperative management [38]. Finally, smoking is highly prevalent in patients with bladder cancer and RCC, both of which are among the most common smoking-related human malignancies [37]. Likewise, in this study, 57% were former or current smokers in the RCC group, and 72% were former or current smokers in the UTUC group, making smoking cessation interventions a necessity in future preoperative strategies. 

Collectively, these findings support a precision prehabilitation approach, tailored to the individual’s baseline deficits in physical function, nutrition, and resilience. The impaired physical function at baseline, the postoperative decline in function, and the incomplete recovery observed at follow-up underscore a narrow but critical window for intervention.

### Strengths and Limitations

The strengths of this study include its prospective design and the use of objective and validated functional assessments tools. Moreover, this study, to our knowledge, is the first to report a baseline assessment of physical function in patients undergoing surgery for RCC and UTUC cancers. The inclusion of both nutritional and functional screening tools allows for a comprehensive evaluation of modifiable risk factors relevant for possible future prehabilitation interventions. Additionally, assessments were conducted longitudinally at multiple perioperative time points, providing insight into the postoperative changes in physical function over the course of treatment. Some limitations due to the design should be acknowledged. The study design was purely observational and not randomized. The short follow-up period of two weeks postoperatively may not reflect longer-term recovery or the potential benefits of prehabilitation interventions; rather, it solely reflects functional status. Despite these limitations, this study highlights critical preoperative vulnerabilities and may promote the development of personalized prehabilitation strategies in surgical treatment of RCC and UTUC.

## 5. Conclusions

This study demonstrated substantial postoperative decline in functional status in patients undergoing nephrectomy for RCC or nephroureterectomy because of UTUC with only partial recovery within two weeks of surgery. The findings also added valuable insight into the functional status prior to surgery and address the need for early identification of high-risk individuals and identification of relevant prehabilitation targets to optimize perioperative care in kidney cancer surgery. Integrating multimodal, individualized prehabilitation strategies into perioperative pathways may improve long-term recovery outcomes.

## Figures and Tables

**Table 1:** Preoperative characteristics of patients undergoing nephrectomy or nephroureterectomy at Aarhus University Hospital from February 2023 to February 2024.

	Total	Nephrectomy	Nephroureterectomy
	N (%) = 62	N_1_ (%) = 31	N_2_ (%) = 31
**Age**	69 (63;79) *	68 (60;74) *	73 (66;81) *
**Gender**			
Women	23 (37%)	9 (29%)	14 (45%)
Men	39 (63%)	22 (71%)	17 (55%)
**Surgical modus**			
-Laparoscopy	39 (63%)	22 (71%)	17 (55%)
-Robot	18 (29%)	4 (13%)	14 (45%)
-open	5 (8%)	5 (16%)	0 (0%)
**Location**			
Right	37 (60%)	19 (61%)	18 (58%)
Left	25 (40%)	12 (39%)	13 (42%)
**Nutritional status (NRS-2002)**			
1—No risk	0 (0%)	0 (0%)	0 (0%)
2—Moderate risk	27 (44%)	17 (55%)	10 (32%)
3—At risk	35 (56%)	14 (45%)	21 (68%)
**BMI (G8)**			
BMI < 18.5	0 (0%)	0 (0%)	0 (0%)
18.5 ≤ BMI < 21	4 (7%)	2 (7%)	2 (7%)
21 ≤ BMI < 23	7 (12%)	2 (7%)	5 (16%)
BMI ≥ 23	47(75%)	23 (74%)	24 (77%)
Missing	4 (6%)	4 (12%)	
**Smoking status**			
Smoker	13 (21%)	4 (14%)	9 (30%)
Former smoker	25 (40%)	12 (43%)	13 (42%)
Never smoked	19 (31%)	12 (43%)	7 (22%)
Missing	5 (8%)	3 (9%)	2 (6%)
**Polypharmacy**	33 (55%)	17 (57%)	16 (53%)
Missing	2	1	1
**Frailty (Clinical frailty Scale)**			
1—Very fit	19 (31%)	11 (35%)	8 (26%)
2—Fit	15 (24%)	7 (23%)	8 (26%)
3—Managing well	16 (26%)	7 (23%)	9 (29%)
4—Living with very mild frailty	10 (16%)	5 (16%)	5 (16%)
>=5—Living with frailty	2 (3%)	1 (3%)	1 (3%)
**Neuro-psychological condition (G8)**			
Severe dementia or depression	0 (0%)	0 (0%)	0 (0%)
Mild dementia	3 (5%)	2 (6%)	1 (3%)
No psychological condition	59 (95%)	29 (94%)	30 (97%)
**Anemia: Hemoglobin (Hgb) levels (G8)**			
Severe anemia (Hgb < 5.6 mmol/L)	1 (2%)	1 (3%)	0 (0%)
Moderate (Hgb 5.6–6.4 mmol/L)	3 (5%)	1 (3%)	2 (7%)
Mild (Hgb > 6.4 mmol/L)	30 (48%)	16 (52%)	14 (45%)
No anemia	28 (45%)	13 (42%)	15 (48%)
**Serum Iron**			
<9 my-mol	27 (44%)	14 (45%)	13 (42%)
>=9;34 my-mol	22 (35%)	11 (35%)	11 (35%)
Missing	13 (21%)	6 (19%)	7 (23%)
**Serum-Albumin**	36.0 (34.0;40.0) *	36.0 (34.0;40.0) *	36.5 (33.3;40.0) *
**Self-perceived health status (G8)**	2 (1;3) *	2 (1;3) *	2 (1;3) *

* median (Q1–Q3).

## Data Availability

The dataset is available on request from the authors. The raw data supporting the conclusions of this article will be made available by the authors on request. All data were anonymized and entered into an external REDCap database under the Central Region of Denmark.

## Abbreviations

The following abbreviations are used in this manuscript:

RCC	Renal Cell Cancer
UTUC	Upper-tract Urothelial Carcinoma
6MWT	Six-Minute Walk Test
30STS	30-Second Sit-to-Stand Test
ERAS	Enhanced Recovery after Surgery
ACROBATIC	The Danish National Center for Research in Cancer Surgery
LLVs	Lower Limit Values
BMI	Body Mass Index
NRS-2002	Nutritional Risk Screening 2002
